# Supplementary material for: Clinical Significance and Systematic Expression Analysis of the Thyroid Receptor Interacting Protein 13 (TRIP13) as Human Gliomas Biomarker
Source: Cancers (Basel). 2021 May 12;13(10):2338. doi: 10.3390/cancers13102338 (PMC8150328; doi:10.3390/cancers13102338)
Supplement: Supplementary file 1 [file cancers-13-02338-s001.zip › cancers-1189133-supple-xml/cancers-1189133-supple figures.pdf]

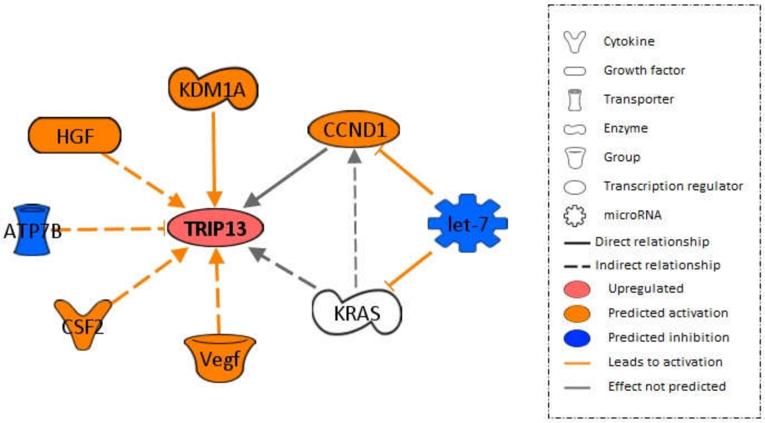

Figure S1. IPA pathway analysis of TRIP13 and putative upstream regulators. Ingenuity Pathway analysis (IPA) identified many upstream regulators predicted activation or inhibition based on the gene expression in TCGA data and knowledge base of IPA. TRIP13 is upregulated by predicted activation state of KDM1A, HGF, CSF2 and VEGF and CCND1, whereas the ATP7B is predicted as inhibited. Let-7 (microRNA) is involved in the network and predicted inhibition, which lead to regulate CCND1 and KRAS, to affect downstream TRIP13 expression. The figure legend describes predicted relationships of TRIP13 gene to the upstream regulators.

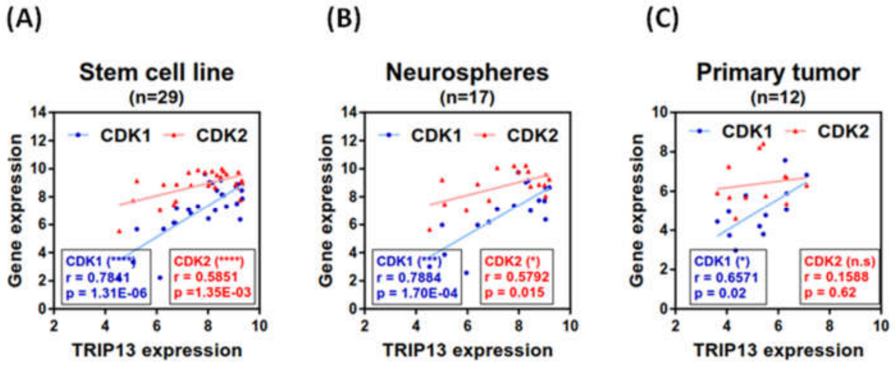

Figure S2. Positive correlation analysis between the transcriptional levels of TRIP13 and DNA damage checkpoint proteins (CDK1 and CK2) in stem cell lines, neurospheres and the corresponding primary tumors from GDS3885 dataset. r, Pearson correlation coefficient. p, *p*-value indicates the significance of the correlation. $*p < 0.05$, $**p < 0.01$, $***p < 0.001$ and $****p < 0.0001$; n.s: not significant.

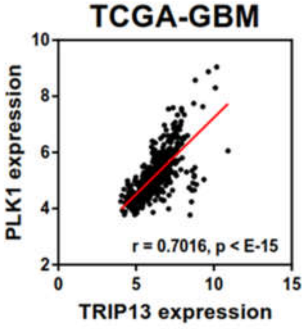

Figure S3. Positive correlation analysis between the transcriptional levels of TRIP13 and PLK1 in TCGA-GBM dataset. r, Pearson correlation coefficient. p, *p*-value indicates the significance of the correlation.

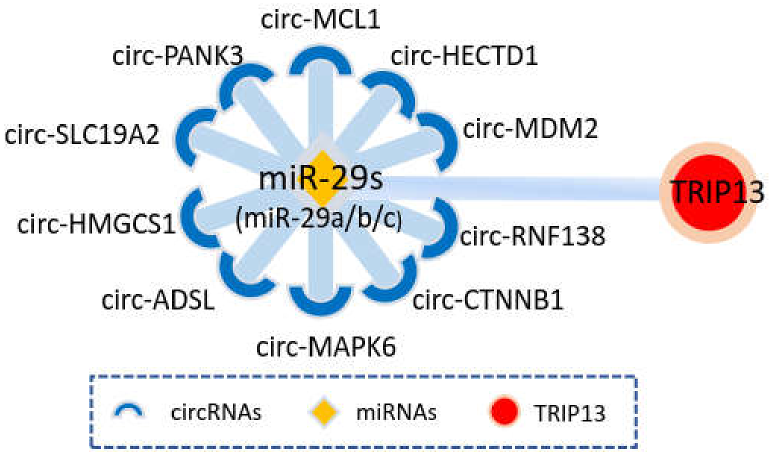

Figure S4. The scheme of circRNA-miRNA-RNA networks. Top 10 circRNAs were identified by glioma samples (obtained from circRNADb human circRNA database) and selected interacting with miR-29a, 29b and 29c by starBase program.

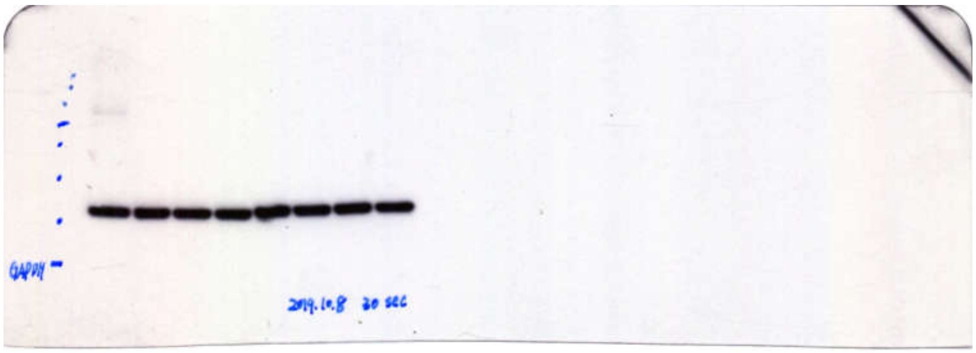

Figure S5. Western blot analysis of the glyceraldehyde-3-phosphate dehydrogenase (GAPDH) as a control for quantitative real-time PCR.

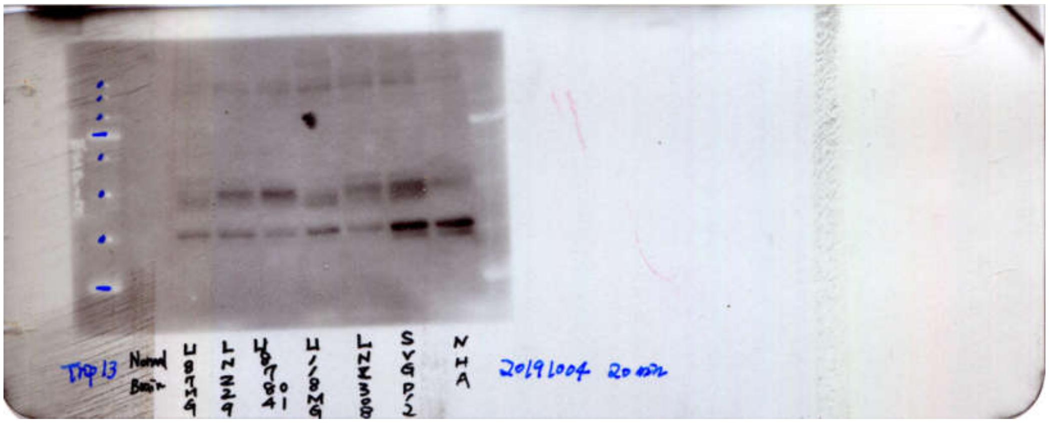

Figure S6. Western blot analysis of TRIP13 expression in normal brain, U87MG, LN229, GBM8401, U118MG, LNZ308, SVGp12, NHA cell lines.
